# The course of swallowing problems in the first 2 years after diagnosis of head and neck cancer

**DOI:** 10.1007/s00520-022-07322-w

**Published:** 2022-08-30

**Authors:** Jorine A. Vermaire, Cornelis P. J. Raaijmakers, Evelyn M. Monninkhof, C. René Leemans, Robert J. Baatenburg de Jong, Robert P. Takes, Irma M. Verdonck-de Leeuw, Femke Jansen, Johannes A. Langendijk, Chris H. J. Terhaard, Caroline M. Speksnijder

**Affiliations:** 1 Department of Radiation Oncology, Imaging Division, University Medical Center Utrecht, Utrecht University, Utrecht, the Netherlands; 2 Department of Epidemiology, Julius Center for Health Sciences and Primary Care, University Medical Center Utrecht, Utrecht University, Utrecht, the Netherlands; 3 Department of Otolaryngology-Head and Neck Surgery and Cancer Center Amsterdam, Amsterdam UMC, Vrije Universiteit Amsterdam, Amsterdam, the Netherlands; 4 Department of Otorhinolaryngology, Head and Neck Surgery, Erasmus MC Cancer Center, Rotterdam, the Netherlands; 5 Department of Otolaryngology-Head and Neck Surgery, Radboud University Medical Center, Nijmegen, the Netherlands; 6 Department of Clinical, Neuro- and Developmental Psychology, Amsterdam Public Health Research Institute, Vrije Universiteit Amsterdam, Amsterdam, the Netherlands; 7 Department of Radiation Oncology, University Medical Center Groningen, Groningen, the Netherlands; 8 Department of Oral and Maxillofacial Surgery and Special Dental Care, University Medical Center Utrecht, Utrecht University, G05.122, P.O. Box 85.500, 3508 GA Utrecht, the Netherlands; 9 Department of Head and Neck Surgical Oncology, University Medical Center Utrecht, Utrecht University, Utrecht, the Netherlands

**Keywords:** Linear mixed-effects model, Head and neck cancer, SWAL-QOL, Oral function, Swallowing

## Abstract

**Introduction:**

Head and neck cancer (HNC) and its treatment often negatively impact swallowing function. The aim was to investigate the course of patient-reported swallowing problems from diagnosis to 3, 6, 12, and 24 months after treatment, in relation to demographic, clinical, and lifestyle factors.

**Methods:**

Data were used of the Netherlands Quality of Life and Biomedical Cohort Study in head and neck cancer research (NET-QUBIC). The primary outcome measures were the subscales of the Swallowing Quality of Life Questionnaire (SWAL-QOL). Linear mixed-effects models (LMM) were conducted to investigate changes over time and associations with patient, clinical, and lifestyle parameters as assessed at baseline.

**Results:**

Data were available of 603 patients. There was a significant change over time on all subscales. Before treatment, 53% of patients reported swallowing problems. This number increased to 70% at M3 and decreased to 59% at M6, 50% at M12, and 48% at M24. Swallowing problems (i.e., longer eating duration) were more pronounced in the case of female, current smoking, weight loss prior to treatment, and stage III or IV tumor, and were more prevalent at 3 to 6 months after treatment. Especially patients with an oropharynx and oral cavity tumor, and patients receiving (C)RT following surgery or CRT only showed a longer eating duration after treatment, which did not return to baseline levels.

**Conclusion:**

Half of the patients with HNC report swallowing problems before treatment. Eating duration was associated with sex, smoking, weight loss, tumor site and stage, and treatment modality, and was more pronounced 3 to 6 months after treatment.

## Introduction

Head and neck cancer (HNC) is the seventh most common cancer worldwide, accounting for an estimated 650,000 new cases and 350,000 deaths every year [1]. HNC is most often caused by alcohol and/or tobacco use, or the human papilloma virus (HPV) [2]. Curative treatment options for HNC include surgery, radiotherapy (RT), and chemoradiation (CRT). Treatment extent and intensity vary, and the choice of treatment modality depends on tumor site, tumor stage, co-morbidities, and wishes and expectations of patients [3, 4]. Surgery may compromise lingual mobility, strength, and muscle coordination in the head and neck region [4–6]. High-intensity radiation treatment regimens have resulted in improved survival and tumor control, but may also lead to acute effects such as pain, mucositis, and decrease in saliva production, and late effects such as trismus, masticatory deficits, dysphagia (swallowing dysfunction), and xerostomia [4, 7, 8]. Chemotherapy can add to these effects by increasing oral mucositis, nausea, vomiting, loss of appetite, and xerostomia [9]. These side effects occur in a considerable proportion of patients after HNC treatment despite efforts to spare structures related to oral food processing, salivary function, and swallowing [7, 10]. During the food process, several muscles, nerves, and connective tissue structures need to work together to break down food into smaller particles which bind to each other through saliva, and form a food bolus ready for swallowing and digestion [11, 12]. The number of teeth and occlusal units are of great importance to grind and break down food. Tooth loss, the presence of cavities, inadequate restorations, malocclusion, or periodontal disease can adversely affect chewing function and thereby also swallowing [13, 14]. Side effects of treatment may have a negative influence on swallowing function and thereby on the ability to eat and drink, which in turn impact health-related quality of life (QOL) of patients [1, 15, 16].

To evaluate patient-reported outcomes (PROs) regarding dysphagia, several tools are available such as the swallowing subscale of the European Organization for Research and Treatment of Cancer Quality of Life Questionnaire (EORTC QLQ-H&N35), the M.D. Anderson Dysphagia Inventory (MDADI) [17, 18], and the Swallowing Quality of Life Questionnaire (SWAL-QOL) [16]. An important study using the EORTC QLQ-H&N35 to assess swallowing (*n* = 2458) provided a survey at baseline, and 4 and 12 months post-baseline [19]. This study included all possible patients with HNC (all curative treatment options and tumor sites). Swallowing was diminished especially 4 months after treatment. Factors associated with swallowing and social eating were as follows: tumor site, age, treatment, smoking, socio-economic status, and sex [19].

Unlike the EORTC QLQ-H&N35 questionnaire (which only has a swallowing and social eating subscale), the SWAL-QOL questionnaire includes multiple subscales to assess swallowing-related quality of life. Multiple studies assessed swallowing as measured with the SWAL-QOL, either as prospective cohort study to investigate swallowing differences over time [7, 20–24] or as cross-sectional study to investigate swallowing at one point in time [10, 21, 22, 25, 26], for example, at baseline [21]. In these studies, different patients were assessed. Some studies only included patients that received RT or CRT [7, 10, 20, 22, 23, 25, 26], other studies only patients that received surgery [24], and others included patients with all curative treatment options [21, 27]. In addition, tumor site differed from one tumor site (e.g., laryngeal [27]) to all tumors in the head and neck region. The number of patients, included in the various studies assessing the SWAL-QOL, varied as well, from 22 [20] to 1083 patients [22]. Most studies found that swallowing function was impaired across most domains for the majority of patients [23, 24, 27], especially 6 to 12 months after treatment [10].

To reduce the risk for persistent patient-reported swallowing problems after treatment for HNC, it is important to identify factors associated with these swallowing problems. Information can be provided about possible problems that may occur after treatment and the possibility of rehabilitation during and after treatment can be discussed. This will lead to a better evaluation of possible treatment options and more patient-centered care. As mentioned, previous research about the SWAL-QOL mainly focused on one type of treatment modality, or one type of tumor site [28, 29]. In addition, most studies investigating swallowing problems in patients with HNC were too small to allow subgroup analyses [30]. One study included all patients with HNC and assessed a large cohort, but only assessed the subscales swallowing and eating duration of the EORTC QLQ-H7N35 [19]. Factors that were found to be associated with poor patient-reported swallowing problems included patient-related factors such as smoking, alcohol use, higher age, low socio-economic status, and being female, and tumor-related factors such as advanced tumor stage, multi-modality treatment, and tumor site [10, 19, 31]. However, to our knowledge, there are no studies that assessed the SWAL-QOL questionnaire and included the majority of these factors, included all treatment modalities and tumor sites, and assessed swallowing problems prospectively up to 2 years after treatment.

The primary aim of this study was to investigate the course over time in the first 2 years after HNC diagnosis of various aspects of patient-reported swallowing problems as measured with the SWAL-QOL. The secondary aim was to identify demographic, clinical, and lifestyle factors associated with patient-reported swallowing problems in patients with HNC.

## Materials and methods

Data were used of 739 patients with HNC participating in the prospective NETherlands Quality of Life and Biomedical Cohort study in HNC cancer (NET-QUBIC), of which details were published previously [32, 33]. Recruitment took place in 7 HNC centers throughout the Netherlands between 2014 and 2018. Patients were included when they were 18 years or older, were diagnosed with oral, oropharyngeal, hypopharyngeal, or laryngeal HNC, and were treated with curative intent (all treatment modalities). Patients with an unknown primary tumor, recurrent or residual disease, cognitive impairments, lymphoma, skin malignancies, or thyroid cancer, and patients having trouble understanding or reading the Dutch language were excluded. All patients signed written informed consent before participation. The study protocol of this prospective observational cohort study was approved by the Medical Ethics Committee of the VUmc (NL45051.029.13) and all local participating centers [32, 33]. In the present study, patients were included when they had completed the SWAL-QOL questionnaire at any given time point. The SWAL-QOL questionnaire was assessed before primary treatment (baseline, M0), 3 months (M3), 6 months (M6), 12 months (M12), and 24 months after treatment (M24). Demographic factors (age and sex), clinical factors (tumor stage [34], tumor site, HPV status (in oropharynx patients), treatment modality, comorbidity, and weight loss), and lifestyle factors (alcohol use and smoking) were assessed at baseline.

The primary outcome measure was the 47-item Swallowing Quality of life Questionnaire (SWAL-QOL) [16]. This questionnaire comprises of 10 subscales on food selection (2 items), eating duration (2 items), eating desire (3 items), fear of eating (4 items), general burden (2 items), mental health (5 items), social functioning (5 items), communication (2 items), sleep (2 items), and fatigue (3 items). Furthermore, a symptom scale (14 items) is included. Based on the 23 items of the first seven mentioned scales, a total SWAL-QOL score can be calculated. All items refer to the last month. In NET-QUBIC, the subscales communication, sleep, and fatigue were removed, because of the considerable overlap with the Speech Handicap Index and the Multidimensional Fatigue Inventory. The 5-point items are transformed to scales ranging from 0 to 100, where a higher score indicates more swallowing problems. As found in previous research, a cut-off score on the total SWAL-QOL score of ≥ 14 points indicates a high level of swallowing problems in daily life [26]. The SWAL-QOL has been translated into Dutch and validated for use in patients with HNC [16].

Baseline characteristics about age, sex, ACE-27 comorbidity score [35], TNM7 classification (2010), and weight loss prior to treatment were collected from medical files. HPV status was collected for oropharynx tumors. A 13-item study-specific patient-reported questionnaire was used to assess smoking status and nicotine dependence. One item about passive smoking was included, 7 items about smoking behavior, and 5 items about nicotine dependence. For this study, patients were categorized as current smoker, nonsmoker (less than 100 units in their lifetime), or former smoker. A 21-item questionnaire was used to assess alcohol intake and dependence, consisting of questions about current alcohol intake and history of alcohol intake (14 items), and alcohol dependence (7 items). The question “do you drink regularly” was used to assess alcohol intake in the current study.

### Statistical analyses

Descriptive statistics were used to describe the study population. Differences between the total NET-QUBIC population and patients that filled in the SWAL-QOL were tested using ANOVA to assess differences in age, and chi-square tests were run to test for differences in sex, tumor site, tumor stage, primary treatment, alcohol consumption, smoking, comorbidity, and weight loss.

Linear mixed models (LMM) were used to assess if demographic, clinical, and lifestyle factors influenced changes over time of the total score and all subscales of the SWAL-QOL. Akaike’s Information Criterion (AIC) was used to select the most appropriate covariance structure to fit the data [36]. To account for within-patient correlations, a random patient factor was added, and a random intercept was used to account for the different entry levels of patients. The fixed-effect factors timing of assessment, tumor site, treatment modality, tumor stage based on TNM classification [34], sex, alcohol consumption, smoking, comorbidity, weight loss, HPV status, and age, as well as 2-way interactions of the factors treatment modality, tumor site, and tumor stage during the assessment period, were assessed. Timing of assessment consisted of 5 levels (M0, M3, M6, M12, M24), tumor site consisted of 3 levels (oropharynx, larynx or hypopharynx, oral cavity), treatment modality consisted of 4 levels (RT, CRT, surgery or CO_2_ laser, surgery with post-operative (C)RT), tumor stage consisted of 4 levels (I:Tis or T1N0M0, II:T2N0M0, III:T3N0M0 or T2N1M0 or T3N1M0, IV:T4aN0M0 or TanyN2M0 or T4bN0M0 or TanyN3M0) [34], sex consisted of 2 levels (male, female), alcohol consumption consisted of 3 levels (drink regularly, seldom drink, drank in the past), smoking consisted of 3 levels (nonsmoker, former smoker, smoker), comorbidity consisted of 4 levels (none, mild, moderate, severe), weight loss consisted of 3 levels (no weight loss, 1–5 kg weight loss, > 5 kg weight loss), HPV status consisted of 2 levels for oropharynx patients only (positive, negative), and age was defined as a continuous variable. The model included a stepwise backward selection of factors, in which factors that were not significant at a *p* < 0.10 level were removed, beginning with the interactions. A hierarchical structure was maintained, meaning that if an interaction was included in the model, the main effects were also represented in the model. Risk factors were reported as estimated unstandardized regression coefficients with 95% confidence intervals (CI) and *p*-values.

The coefficients of the significant covariates, together with the value of the intercept of the mixed model analysis, were combined into a formula for the estimated SWAL-QOL subscale. The intercept is the value of the estimated SWAL-QOL subscale in which all coefficients remain zero. Addition of the coefficients will lead to an increase or decrease of the estimated SWAL-QOL subscale. This formula can be used to estimate the QOL of patients during the follow-up period. For each time point, the formula was filled with average variable values for significant coefficients, as calculated by a restricted maximum likelihood approach. All analyses were performed using Statistical Package for the Social Sciences (SPSS) version 25 (Chicago, IL). *p*-values < 0.05 for the descriptive statistics and < 0.10 for the linear mixed-effects model were considered statistically significant.

## Results

**Fig. 1:**
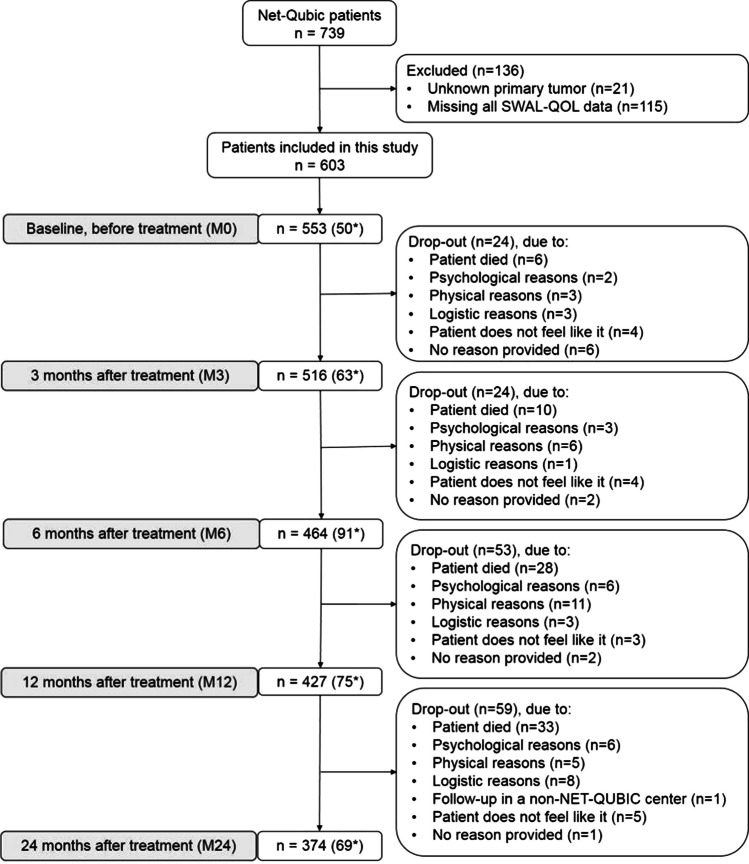
Flowchart depicting the number of patients at each time point. *Missing measurement

Among the 739 patients participating in the NET-QUBIC research, 603 patients filled in the questionnaire at least once during the 2-year follow-up and were included in the LMM analyses. At baseline, 553 patients completed the SWAL-QOL questionnaire, 516 at 3 months, 464 at 6 months, 427 at 12 months, and 374 at 24 months (Fig. 1). No significant differences were observed between patients that responded to the SWAL-QOL questionnaire and the total NET-QUBIC population (Table 1). Based on the SWAL-QOL cut-off score of ≥ 14, 53% of patients had problems before treatment, which increased to 70% at 3 months after treatment and decreased to 59% at 6 months after treatment, 50% at 12 months after treatment, and 48% at 24 months after treatment.

**Table 1:** Characteristics of patients that were included in the NET-QUBIC study, patients included in the linear mixed model analyses, and patients that responded to the SWAL-QOL questionnaire (responders) at baseline (M0)

Variables	Total NET-QUBIC study *n* = 739 *n* (%)	Patients included in LMM analyses *n* = 603 *n* (%)	Responders M0 *n* = 553 *n* (%)	*p*-value	
				Total NET-QUBIC study versus patients included in LMM analyses	Total NET-QUBIC study versus responders M0
Age mean (SD)	63.3 (9.7)	63.5 (9.5)	63.5 (9.6)	0.602†	0.637†
Sex				0.943ǂ	0.860ǂ
Male	549 (74.3)	449 (74.5)	410 (74.1)		
Female	190 (25.7)	154 (25.5)	143 (25.9)		
Tumor site				0.927ǂ	0.837ǂ
Oropharynx	262 (35.5)	217 (36.0)	201 (36.3)		
HPV positive	130 (49.6)	114 (52.5)	106 (52.7)	0.425ǂ	0.679ǂ
HPV negative	99 (37.8)	74 (34.1)	68 (33.8)		
Missing	*33 (12.6)*	*29 (13.4)*	27 (13.4)		
Larynx or hypopharynx	257 (34.8)	210 (34.8)	191 (34.5)		
Oral cavity	199 (26.9)	176 (29.2)	161 (29.1)		
Unknown primary	*21 (2.8)*	*0*	*0*		
Tumor stage				0.562ǂ	0.715ǂ
1	163 (22.1)	150 (24.9)	139 (25.1)		
2	132 (17.9)	113 (18.7)	105 (19.0)		
3	127 (17.2)	98 (16.3)	88 (15.9)		
4	317 (42.9)	242 (40.1)	221 (40.0)		
Primary treatment				0.831ǂ	0.901ǂ
RT	241 (32.6)	199 (33.0)	189 (34.2)		
CRT	215 (29.1)	163 (27.0)	147 (26.6)		
Surgery or CO_2_ laser	152 (20.6)	133 (22.1)	121 (21.9)		
Surgery with PO(C)RT	129 (17.4)	107 (17.7)	96 (17.3)		
Other	*2 (0.3)*	*1 (0.2)*	*0*		
Alcohol consumption				0.999ǂ	0.991ǂ
Drink regularly	313 (42.4)	302 (50.1)	298 (53.9)		
Seldom drink	170 (23.0)	164 (27.2)	164 (29.7)		
Drank in past	88 (11.9)	85 (14.1)	85 (15.4)		
Missing	*168 (22.7)*	*52 (8.6)*	*6 (1.0)*		
Smoking				0.997ǂ	0.982ǂ
Nonsmoker	105 (14.2)	101 (16.7)	100 (18.1)		
Former smoker	321 (43.4)	311 (51.6)	309 (55.9)		
Smoker	146 (19.7)	140 (23.2)	139 (25.1)		
Missing	*168 (22.7)*	*51 (8.5)*	*5 (0.9)*		
Comorbidity				0.907ǂ	0.731ǂ
None	204 (27.6)	177 (29.4)	164 (29.7)		
Mild	264 (35.7)	218 (36.2)	203 (36.7)		
Moderate	155 (21.0)	119 (19.7)	108 (19.5)		
Severe	76 (10.3)	61 (10.1)	52 (9.4)		
Missing	*40 (5.4)*	*28 (4.6)*	*26 (4.7)*		
Weight loss				0.945ǂ	0.745ǂ
No weight loss	471 (63.7)	386 (64.0)	357 (64.6)		
1–5 kg	121 (16.4)	96 (15.9)	88 (15.9)		
> 5 kg	71 (9.6)	55 (9.1)	48 (8.7)		
Missing	*76 (10.3)*	*66 (11.0)*	*60 (10.8)*		

†ANOVA; ǂchi-square

*CRT* chemoradiation, *LMM* linear mixed model, *PO* post-operative, *RT* radiotherapy, *SD* standard deviation, *SWAL-QOL* Swallowing Quality of Life Questionnaire

### Mean SWAL-QOL outcomes

**Fig. 2:**
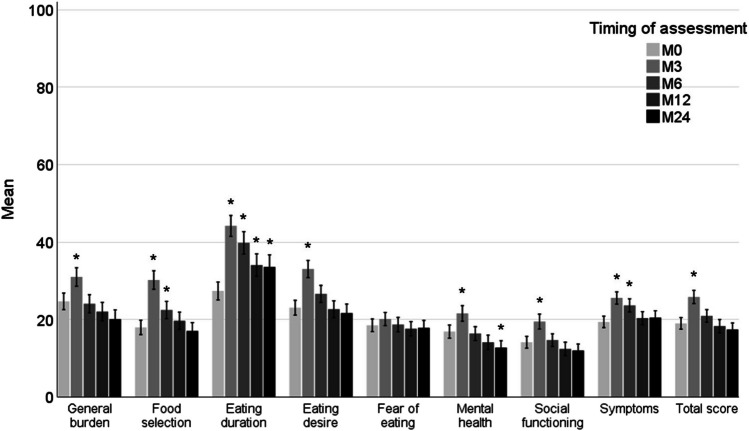
Mean scores of the SWAL-QOL total score and subscales with standard error from baseline (M0) up to 2 years (M24) after treatment in patients with head and neck cancer. *p < 0.05 in comparison to baseline. SWAL-QOL, Quality of Life in Swallowing Disorders questionnaire

The total score and subscales “general burden,” “eating desire,” and “social functioning” showed higher mean scores for all patients 3 months after treatment (indicating more problems), after which these scores returned to baseline at 6 months after treatment and beyond (Fig. 2). There was no change over time in “fear of eating.” The subscales “food selection” and “symptoms” took longer to return to baseline levels, indicated by the significant differences at 6 months after treatment. “Mental health” was higher 3 months after treatment and lower at 24 months after treatment in comparison to baseline. “Eating duration” increased from baseline to 3 months after treatment, remained significantly worse 6 months after treatment, and did not return to baseline 12 and 24 months after treatment. Because the total score and all subscales of the SWAL-QOL returned to baseline, except the subscale “eating duration,” the focus of the subsequent linear mixed model was on this subscale.

### Eating duration evaluated with linear mixed model

**Fig. 3:**
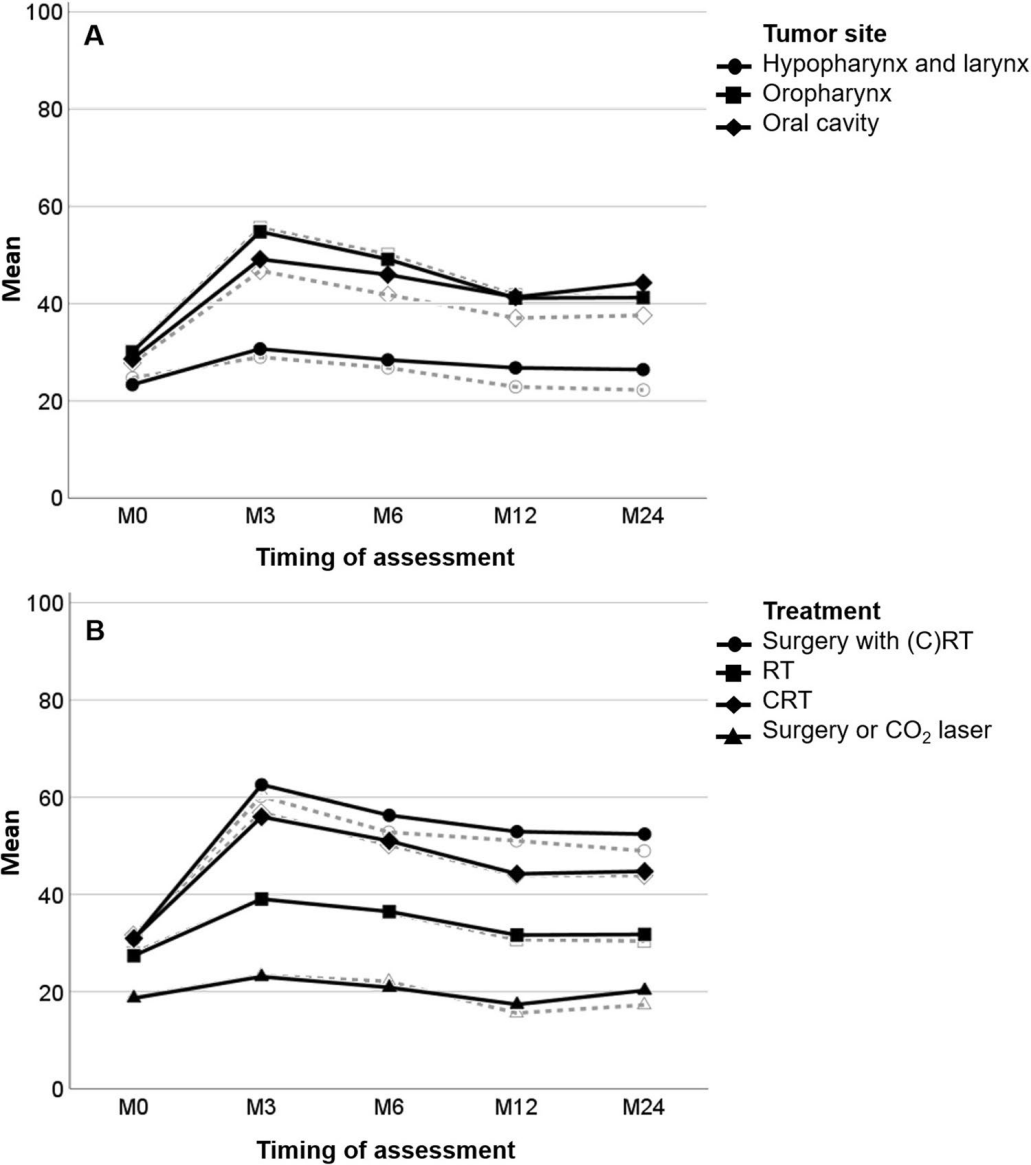
The mean outcomes for eating duration based on tumor site (**A**) and treatment modality (**B**) to provide insight in the raw and modeled data. The solid lines represent the linear mixed model outcomes of the final model; the striped lines represent the raw data. CRT, chemoradiation; RT, radiotherapy

The factors alcohol, comorbidity, HPV status, age, and the interaction between timing of assessment and tumor stage were not associated with eating duration and were therefore removed from the model. The factors sex, smoking, weight loss, tumor site, treatment, tumor stage, and timing of assessment were associated with a worse eating duration (Table 2). The eating duration was worse in females in comparison to males (+ 4.15). Furthermore, smoking (+ 11.23 in comparison to nonsmokers) and losing weight before treatment (+ 7.56 for > 5 kg in comparison to no weight loss), and more advanced tumor stage (stages III–IV) (+ 11.01 for stage IV in comparison to stage I) [34], were associated with a worse eating duration. The eating duration increased from baseline to 3 months after treatment (+ 8.15), remained significantly worse 6 months after treatment (+ 6.67), and did not return to baseline 12 and 24 months after treatment (+ 1.33 and + 6.11, respectively). In addition, significant interactions were found for timing of assessment with tumor site and treatment, indicating that the course over time differed between different tumor sites and different treatment modalities (Fig. 3). Patients with a tumor located in the oropharynx or oral cavity showed a worsening in eating duration 3 and 6 months after treatment, after which the numbers did not return to baseline 12 and 24 months after treatment but remained ± 10 points higher (Fig. 3A). Patients with a tumor in the hypopharynx or larynx did not show a worsening in eating duration after treatment; instead, the scores remained constant over time. Patients receiving adjuvant (C)RT and patients receiving definitive CRT only showed the worst decline in outcomes from baseline to 3 months after treatment. These numbers did not return to baseline but remained high from 6 to 24 months after treatment. Patients receiving RT only showed a mild worsening from baseline up to 3 months after treatment, after which the numbers slowly returned to baseline. Patients receiving surgery only or CO_2_ laser treatment (for early laryngeal cancers) showed no decline after treatment.

**Table 2:** Linear mixed model estimates for the subscale eating duration of the SWAL-QOL questionnaire

Characteristic		Estimate (95% CI)	*p*-value	Interactions with timing of assessment									
				M0		M3		M6		M12		M24	
Intercept		47.66 (36.56 to 58.75)		Estimate (95% CI)	*p*-value	Estimate (95% CI)	*p*-value	Estimate (95% CI)	*p*-value	Estimate (95% CI)	*p*-value	Estimate (95% CI)	*p*-value
Sex	Male	− 4.15 (− 8.78 to 0.48)	0.079*										
	Female	*Reference*											
Weight loss	No weight loss	− 7.56 (− 14.57 to − 0.55)	0.035*										
	1–5 kg	− 0.10 (− 8.25 to 8.04)	0.980										
	> 5 kg	*Reference*											
Smoking	Nonsmoker	− 11.23 (− 17.53 to − 4.93)	0.001*										
	Former smoker	− 8.01 (− 12.82 to − 3.19)	0.001*										
	Smoker	*Reference*											
Tumor stage	I	− 11.01 (− 18.01 to − 4.00)	0.002*										
	II	− 7.72 (− 14.38 to − 1.06)	0.023*										
	III	2.39 (− 3.90 to 8.68)	0.457										
	IV	*Reference*											
Timing of Assessment	M0	*Reference*											
	M3	8.15 (2.39 to 19.92)	0.006*										
	M6	6.67 (− 0.20 to 13.53)	0.057*										
	M12	1.33 (− 5.97 to 8.63)	0.721										
	M24	6.11 (− 1.50 to 13.72)	0.116										
Tumor site	Hypopharynx or larynx	− 8.56 (− 17.24 to 0.12)	0.053*	*Reference*		− 10.97 (− 18.71 to − 3.24)	0.005*	− 12.84 (− 21.99 to − 3.69)	0.006*	− 7.35 (− 17.15 to 2.44)	0.141	− 12.59 (− 22.81 to − 2.36)	0.016*
	Oropharynx	− 5.35 (− 14.31 to 3.0.61)	0.242	*Reference*		0.50 (− 7.67 to 8.67)	0.904	− 4.30 (− 14.01 to 5.42)	0.386	− 5.18 (− 15.57 to 5.21)	0.328	− 9.81 (− 20.65 to 1.04)	0.076*
	Oral cavity	*Reference*		*Reference*									
Treatment	CO_2_ laser or surgery	*Reference*		*Reference*									
	RT	6.82 (− 2.30 to 15.95)	0.142	*Reference*		9.95 (2.08 to 17.82)	0.013*	11.78 (2.46 to 21.10)	0.013*	9.32 (− 0.61 to 19.25)	0.066*	9.58 (− 0.71 to 19.86)	0.068*
	CRT	2.93 (− 7.56 to 13.42)	0.584	*Reference*		18.79 (10.15 to 27.43)	< 0.001*	19.40 (9.24 to 29.56)	< 0.001*	17.46 (6.67 to 28.25)	0.002*	17.85 (6.58 to 29.12)	0.002*
	Surgery with PO(C)RT	2.77 (− 6.17 to 11.71)	0.543	*Reference*		24.28 (16.67 to 31.89)	< 0.001*	20.64 (11.59 to 29.69)	< 0.001*	22.39 (12.56 to 32.22)	< 0.001*	18.48 (8.14 to 28.82)	< 0.001*

*p < 0.10; *CI* confidence interval, *CRT* chemoradiation, *PO* post-operative, *RT* radiotherapy, *SWAL-QOL* Swallowing Quality of Life Questionnaire

Estimated eating duration = 47.66 − 4.15 male − 7.56 no weight loss − 0.10 (1–5 kg weight loss) − 11.23 nonsmoker − 8.01 former smoker − 11.01 tumor stage1 − 7.72 tumor stage 2 + 2.39 tumor stage 3 + 8.15 M3 + 6.67 M6 + 1.33 M12 + 6.11 M24 − 8.56 hypopharynx & larynx − 5.35 oropharynx + 6.82 RT + 2.93 CRT + 2.77 surgery & (C)RT − 10.97 hypopharynx & larynx*M3 − 12.84 hypopharynx & larynx*M6 − 7.35 hypopharynx & larynx*M12 − 12.59 hypopharynx & larynx*M24 + 0.50 oropharynx*M3 − 4.30 oropharynx*M6 − 5.18 oropharynx*M12 − 9.81 oropharynx*M24 + 9.95 RT*M3 + 11.78 RT*M6 + 9.32 RT*M12 + 9.58 RT*M24 + 18.79 CRT*M3 + 19.40 CRT*M6 + 17.46 CRT*M12 + 17.85 CRT*M24 + 24.28 surgery & (C)RT*M3 + 20.64 surgery & (C)RT*M6 + 22.39 surgery & (C)RT*M12 + 18.48 surgery & (C)RT*M2

### LMM total score and other subscales

The LMM analyses revealed that the total score and the subscales “general burden,” “food selection,” “eating desire,” “fear of eating,” “mental health,” “social functioning,” and “symptoms” scored worse when the patient was a smoker at baseline, had more comorbidities at baseline, and received CRT or surgery followed by (C)RT. In addition, receiving CRT or surgery followed by (C)RT led to more deterioration shortly after treatment. Having a more advanced tumor stage (stages III and IV) also resulted in a worse outcome for the total score and subscales “general burden,” “food selection,” “eating desire,” “fear of eating,” “mental health,” and “symptoms.” Having a tumor in the oral cavity led to worse outcomes on the total score and subscales “general burden,” “food selection,” “eating desire,” “mental health,” “social functioning,” and “symptoms.” Losing weight before treatment resulted in a worse outcome on the total score and subscales “general burden,” “social functioning,” and “symptoms.” Patients with a higher age showed a worse outcome on the subscales “food selection” and “eating desire.” Drinking alcohol regularly led to less problems on the subscales “mental health” and “social functioning.”

## Discussion

This large 2-year prospective cohort study (*n* = 603) identified factors associated with worse swallowing as measured with the SWAL-QOL in patients with HNC and all treatment modalities. In this study, it was shown that patient-reported problems with swallowing increased from baseline to 3 months after treatment, and slowly decreased from 6 months onwards with return to baseline levels at 2 years after treatment in patients with HNC. Based on the SWAL-QOL cut-off score of ≥ 14, which indicates swallowing problems in daily life, 53% of patients had problems before treatment, which increased to 70% at 3 months after treatment and decreased to 59% at 6 months after treatment, 50% at 12 months after treatment, and 48% at 24 months after treatment. After treatment, the subscale eating duration showed the most problems and did not return to baseline. Therefore, this subscale was used in a LMM to identify factors associated with a worse eating duration, to indicate which patients could benefit from preventive strategies and rehabilitation during and after treatment. Eating duration was associated with sex, smoking, weight loss, tumor site, treatment, tumor stage, and timing of assessment. In addition, the interactions of timing of assessment with tumor site and treatment modality were significant, indicating that the course over time differed for different tumor sites and different treatment modalities.

### Comparison with literature

Based on a cut-off score on the total SWAL-QOL score of ≥ 14 points [26], a previous cross-sectional study of patients with HNC (*n* = 52) found a deviant score in 79% of patients, which is higher than the 70% found in this research at 3 months after treatment. One explanation could be that almost 60% of the patients in that study were treated with 3D conformal RT, in which salivary glands were not spared. After 2005, IMRT was introduced, enabling a significant reduction of dose to the salivary glands [26]. Since then, IMRT has been further optimized, sparing, e.g., parotid glands, pharyngeal constrictor muscles, and the supraglottic larynx [37]. Another cross-sectional study with healthy controls (*n* = 111, mean age = 56 years, 44% male) showed that mean scores of all subscales were between 3.7 (social functioning) and 10.4 (fear of eating) [38]. Although these healthy controls were slightly younger and a higher percentage of females responded to the questions, it strongly indicates that most of the patients with HNC already experience swallowing problems before treatment (Fig. 2), and that these problems remain, even 2 years after treatment.

A prospective cohort study from 2021 investigated factors associated with swallowing and social eating (*n* = 2458) as measured with the EORTC QLQ-H&N35, and found that multi-modality treatment, oropharynx tumors, age, sex, living alone, low socio-economic status, and smoking were outcome predictors [19], which were also found in the current study. Another prospective cohort study from 2009 investigated factors associated with swallowing problems as measured with the SWAL-QOL after curative RT in HNC (*n* = 529), and showed in their multivariate analysis that T3-T4 HNC tumors, bilateral irradiation, weight loss, oropharynx tumors, accelerated RT, and concomitant CRT were related to a worse outcome [7]. Besides the factors bilateral irradiation and accelerated RT, which were not part of the current study, the factors are similar to those found in the current study with respect to eating duration. Another prospective cohort study (*n* = 587) from 2016 found the following factors to be associated with less HNC symptoms: older age, higher education, private insurance, no current tobacco use, alcohol use, no comorbidities, early stage cancer, and no current feeding tube [31]. No other studies reported a positive effect of older age regarding HNC symptoms. A cross-sectional study (*n* = 52) investigating tumor site and RT technique in a multivariable regression analysis found that only tumor site was significantly associated with total SWAL-QOL score [26]. Another cross-sectional study (*n* = 110) in patients receiving RT or CRT found that advanced tumors, patients receiving CRT, use of a nasogastric tube, tracheotomy, and continuation of smoking and drinking alcohol decreased QOL [10].

The effect of smoking on treatment outcome has been described in several studies, in which it is known that survival rates are lower and recurrence rates are higher in patients who continue to smoke in comparison to patients who stop smoking [39, 40]. In addition, smokers are at higher risk for treatment failure, disease recurrence, and development of second primary tumors [41]. Smokers showed a poorer response to RT, and increased toxicity and side effects from RT [42]. After surgery, smokers showed significantly higher rates of wound complications and general morbidity, and had an increased risk of infection [43]. In the current study, patients who smoked at baseline reported more swallowing problems in comparison to nonsmokers. Smoking cessation may therefore not only be important for survival and disease recurrence, but also reduce swallowing problems before and after treatment. Besides smoking, it is known that the frequency and severity of swallowing problems are more pronounced when patients lose weight pretreatment (possibly because of the tumor), and that swallowing problems increase when weight loss increases [21]. These effects were also found in the current study, where patients who had lost weight at time of diagnosis experienced more problems in comparison to patients who had no weight loss prior to treatment. It is important that patients receive a nutritional assessment or even undergo placement of a feeding tube during treatment to maintain a healthy weight, and to minimize patient-reported problems in the long term [21, 44].

### Strengths and limitations

The strengths of this study were the prospective study design, the large number of patients, and the use of the LMM checklist with recommendations for reporting multilevel data and analyses [45]. Because only 35 patients received CO_2_ laser treatment, and these results were comparable to the results of patients that received surgery, it was decided to combine these groups. In addition, patients with a larynx and hypopharynx tumor were combined as well (*n* = 205 and *n* = 52 in the total NET-QUBC population, respectively). A limitation of this study was the fact that only 374 patients filled in the questionnaire 2 years after treatment [32, 33]. The 739 patients that were included in the NET-QUBIC research are already a selection of the total HNC population, in which it is unknown whether the non-responders perform worse or better regarding swallowing problems. In addition, there was a relative large group of patients with missing measurements at each time point (Fig. 1). Another limitation of this study was that information about rehabilitation during or after treatment was not taken into account. In addition, there is a low correlation between SWAL-QOL outcomes and objective swallowing functioning tests, as explored in previous research [46]. Therefore, the results found in the current research cannot be translated to objective swallowing performance.

## Conclusion

Patients with HNC reported an increase in swallowing problems from baseline to 3 months after treatment, and a slow decrease from 6 months onwards with return to baseline level at 12 and 24 months. The subscale eating duration of the SWAL-QOL showed the most problems after treatment. A longer eating duration was associated with female sex, smoking and weight loss at time of diagnosis, having tumor stage III or IV, and being 3 to 6 months after treatment. Especially patients with an oropharynx and oral cavity tumor showed a persistent increase in eating duration. In addition, patients receiving (C)RT following surgery and patients receiving CRT only showed the worst decline in outcomes, which did not return to baseline levels after treatment.
