# Correction to: The course of swallowing problems in the first 2 years after diagnosis of head and neck cancer

**DOI:** 10.1007/s00520-022-07355-1

**Published:** 2022-09-10

**Authors:** Jorine A. Vermaire, Cornelis P. J. Raaijmakers, Evelyn M. Monninkhof, C. René Leemans, Robert J. Baatenburg de Jong, Robert P. Takes, Irma M. Verdonck-de Leeuw, Femke Jansen, Johannes A. Langendijk, Chris H. J. Terhaard, Caroline M. Speksnijder

**Affiliations:** 1 Department of Radiation Oncology, Imaging Division, University Medical Center Utrecht, Utrecht University, Utrecht, the Netherlands; 2 Department of Epidemiology, Julius Center for Health Sciences and Primary Care, University Medical Center Utrecht, Utrecht University, Utrecht, the Netherlands; 3 Department of Otolaryngology-Head and Neck Surgery and Cancer Center Amsterdam, Amsterdam UMC, Vrije Universiteit Amsterdam, Amsterdam, the Netherlands; 4 Department of Otorhinolaryngology, Head and Neck Surgery, Erasmus MC Cancer Center, Rotterdam, the Netherlands; 5 Department of Otolaryngology-Head and Neck Surgery, Radboud University Medical Center, Nijmegen, the Netherlands; 6 Department of Clinical, Neuro- and Developmental Psychology, Amsterdam Public Health Research Institute, Vrije Universiteit Amsterdam, Amsterdam, the Netherlands; 7 Department of Radiation Oncology, University Medical Center Groningen, Groningen, the Netherlands; 8 Department of Oral and Maxillofacial Surgery and Special Dental Care, University Medical Center Utrecht, Utrecht University, G05.122, P.O. Box 85.500, 3508 GA Utrecht, the Netherlands; 9 Department of Head and Neck Surgical Oncology, University Medical Center Utrecht, Utrecht University, Utrecht, the Netherlands

**Correction to: Supportive Care in Cancer**

**https://doi.org/10.1007/s00520-022-07322-w**

The correct Fig. 2 is the below:
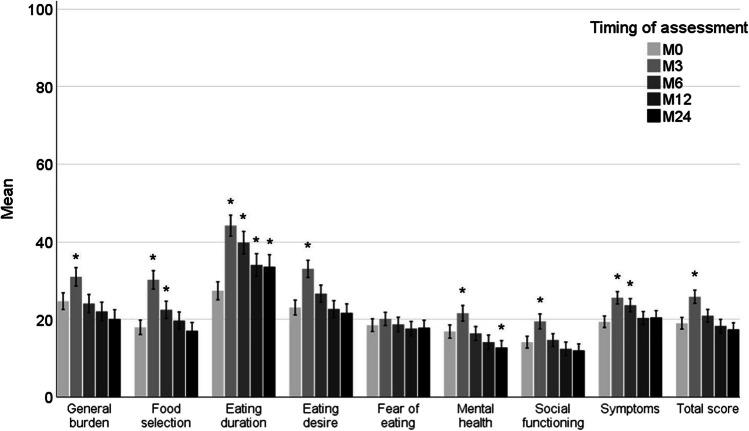


The original article has been corrected.

